# Automated 3D liver segmentation from hepatobiliary phase MRI for enhanced preoperative planning

**DOI:** 10.1038/s41598-023-44736-w

**Published:** 2023-10-17

**Authors:** Namkee Oh, Jae-Hun Kim, Jinsoo Rhu, Woo Kyoung Jeong, Gyu-seong Choi, Jong Man Kim, Jae-Won Joh

**Affiliations:** 1 Department of Surgery, Samsung Medical Center, Sungkyunkwan University School of Medicine, 81 Irwon-ro, Gangnam-gu, Seoul, 06351 Republic of Korea; 2 Department of Radiology and Center for Imaging Sciences, Samsung Medical Center, Sungkyunkwan University School of Medicine, 81 Irwon-ro, Gangnam-gu, Seoul, 06351 Republic of Korea

**Keywords:** Hepatology, Liver

## Abstract

Recent advancements in deep learning have facilitated significant progress in medical image analysis. However, there is lack of studies specifically addressing the needs of surgeons in terms of practicality and precision for surgical planning. Accurate understanding of anatomical structures, such as the liver and its intrahepatic structures, is crucial for preoperative planning from a surgeon’s standpoint. This study proposes a deep learning model for automatic segmentation of liver parenchyma, vascular and biliary structures, and tumor mass in hepatobiliary phase liver MRI to improve preoperative planning and enhance patient outcomes. A total of 120 adult patients who underwent liver resection due to hepatic mass and had preoperative gadoxetic acid-enhanced MRI were included in the study. A 3D residual U-Net model was developed for automatic segmentation of liver parenchyma, tumor mass, hepatic vein (HV), portal vein (PV), and bile duct (BD). The model’s performance was assessed using Dice similarity coefficient (DSC) by comparing the results with manually delineated structures. The model achieved high accuracy in segmenting liver parenchyma (DSC 0.92 ± 0.03), tumor mass (DSC 0.77 ± 0.21), hepatic vein (DSC 0.70 ± 0.05), portal vein (DSC 0.61 ± 0.03), and bile duct (DSC 0.58 ± 0.15). The study demonstrated the potential of the 3D residual U-Net model to provide a comprehensive understanding of liver anatomy and tumors for preoperative planning, potentially leading to improved surgical outcomes and increased patient safety.

## Introduction

Medical image analysis employing deep learning has experienced significant advancements in recent years, as demonstrated by the increasing number of Food Drug Administration (FDA)-approved artificial intelligence (AI)-based diagnostic algorithms^1^. These algorithms have exhibited considerable improvements in accuracy and efficiency for various medical imaging tasks such as diagnosis, segmentation, and quantification when they are assessed from a radiologist’s perspective^2^.

However, from a surgeon’s standpoint, medical images serve as the primary source of information for preoperative planning. Precise understanding of the anatomical structure is crucial for a successful procedure^3^. While medical imaging techniques such as magnetic resonance image (MRI) and computed tomography (CT) scan provide two-dimensional representations of the actual three-dimensional anatomy, it is essential for surgeons to comprehend the anatomy in its entirety^4^. Nevertheless, not all surgeons are adept at converting 2D images to 3D. Even experienced surgeons can be prone to cognitive biases, which may lead to misunderstandings^5^.

This is particularly relevant in the case of the liver, where the tumor’s location and blood vessels within the parenchyma are crucial for determining the appropriate surgical strategy. A more comprehensive understanding of the anatomy can result in improved surgical outcomes and enhanced patient safety by guiding the resection plane and estimating the remnant liver volume^6^. Reconstructing and viewing preoperative images in 3D offer advantage of a complete understanding of the anatomy. This study proposed a deep learning model for automatic segmentation of liver parenchyma, vascular and biliary structures for enhanced surgical planning.

## Results

### Patient demographics

Demographic data such as sex (*p* = 0.458) and age (*p* = 0.741) were not significantly different between train and test sets. The training set included patients having a hepatic mass with various diagnosis, whereas the validation set only included HCC patients, showing no statistically significant difference between the two (*p* = 0.705). Morphological characteristics of tumor such as size (*p* = 0.937), number (*p* = 1.000) and background status of liver (*p* = 0.644) were not significantly different either (Table 1).

**Table 1:** Baseline characteristics of patients.

		Training set	Validation set	*p*
n		108	12	
Sex (%)	Male	86 (79.6)	11 (91.7)	0.458
	Female	22 (20.4)	1 (8.3)	
Age		61.6 ± 11.1	63.0 ± 14.3	0.741
Diagnosis (%)	HCC	80 (74.1)	12 (100.0)	0.705
	HCC + CCC	9 (8.3)	0 (0.0)	
	CCC	7 (6.5)	0 (0.0)	
	CRLM	8 (7.4)	0 (0.0)	
	GB cancer meta	1 (0.9)	0 (0.0)	
	Benign	3 (2.8)	0 (0.0)	
Tumor size (cm, median [IQR])		3.1 [2.2, 4.8]	2.5 [2.2, 5.3]	0.937
Tumor number (%)	1	87 (80.6)	11 (91.7)	1.000
	2	14 (13.0)	1 (8.3)	
	3	5 (4.6)	0 (0.0)	
	4	1 (0.9)	0 (0.0)	
	5	1 (0.9)	0 (0.0)	
Background liver (%)	Normal	54 (50.0)	5 (38.5)	0.644
	Cirrhosis	30 (27.8)	5(38.5)	
	Not reported	24 (22.2)	3 (23.1)	

*HCC* hepatocellular carcinoma, *CCC* cholangiocarcinoma, *CRLM* colorectal cancer liver metastasis, *GB* gall bladder, *IQR* interquartile range.

### Quantitative evaluation

**Figure 1:**
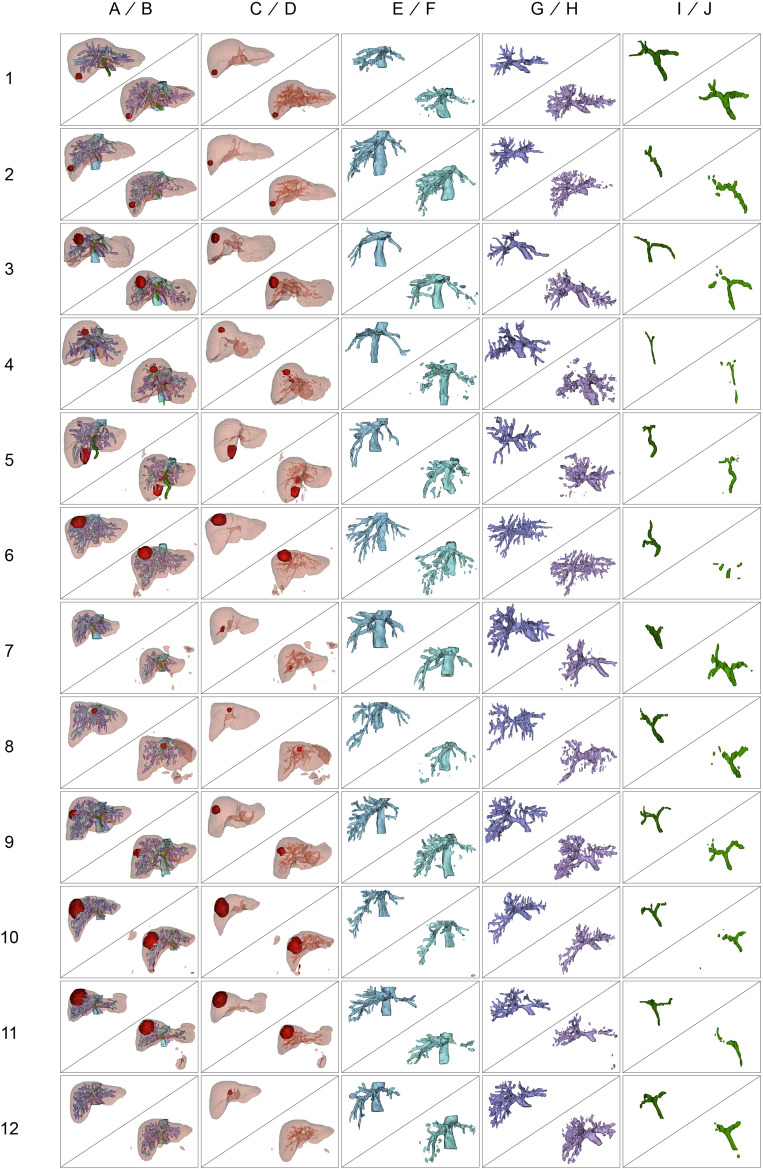
Comparison between manual and automatic segmentation results of the validation set. The 3D reconstructed structures show liver parenchyma (**A** and **B**), tumor mass (**C** and **D**), hepatic vein (**E** and **F**), portal vein (**G** and **H**), and bile duct (**I** and **J**). The manual segmentations are denoted as ground truth (A, C, E, G, I), while the automatic segmentations by the 3D residual U-Net model are denoted as inferred images (B, D, F, H, J). The first column of each row on the y-axis represents the number of patient cases. The images in this figure were created using 3D Slicer v.5.02 (http://www.slicer.org).

Results of manual segmentation and automatic segmentation using 3D residual U-Net model for each case are summarized in Fig. 1, showing 3D reconstructed structures. The mean DSC for the liver parenchyma was found to be the highest at 0.92 ± 0.03, with the best-performing case achieving a DSC of 0.94. The mean DSC for tumor mass was the second highest at 0.77 ± 0.21, with the highest case scoring a DSC of 0.89. The hepatic vein had a mean DSC of 0.70 ± 0.05, with the top-performing case reaching a DSC of 0.77. The portal vein had a mean DSC of 0.61 ± 0.03, with the best case achieving a DSC of 0.65. Finally, the bile duct had the lowest mean DSC of 0.58 ± 0.15, with the highest case obtaining a DSC of 0.76 (Table 2).

**Table 2:** Quantitative comparison of results for each case.

Case	Parenchyma	Tumor	PV	HV	BD
1	0.94	0.78	0.56	0.66	0.76
2	0.94	0.87	0.65	0.77	0.43
3	0.94	0.87	0.64	0.77	0.64
4	0.95	0.88	0.60	0.62	0.50
5	0.93	0.79	0.58	0.69	0.68
6	0.90	0.89	0.65	0.66	0.20
7	0.93	0.74	0.59	0.72	0.64
8	0.90	0.88	0.57	0.63	0.65
9	0.94	0.74	0.58	0.72	0.67
10	0.84	0.86	0.65	0.72	0.57
11	0.88	0.86	0.60	0.71	0.58
12	0.94	0.13	0.63	0.74	0.61
Mean ± SD	0.92 ± 0.03	0.77 ± 0.21	0.61 ± 0.03	0.70 ± 0.05	0.58 ± 0.15

*PV* portal vein, *HV* hepatic vein, *BD* bile duct, *SD* standard deviation.

In addition, vascular and biliary structures were categorized into main and peripheral branches based on the distinction between 1st and 2nd order branches. Then, DSCs were obtained for main and periphery separately, and in all cases, the main branch showed higher DSC values (Table 3).

**Table 3:** DSC for main and peripheral branches of the PV, HV, and BD.

Case	PV	Main	Periphery	HV	Main	Periphery	BD	Main	Periphery
1	0.56	0.75	0.41	0.66	0.78	0.43	0.76	0.83	0.61
2	0.65	0.80	0.50	0.77	0.83	0.53	0.43	0.57	0.00
3	0.64	0.73	0.47	0.77	0.81	0.39	0.64	0.74	0.49
4	0.60	0.76	0.30	0.62	0.75	0.18	0.50	0.65	0.08
5	0.58	0.67	0.44	0.69	0.71	0.53	0.68	0.73	0.33
6	0.65	0.79	0.55	0.66	0.75	0.49	0.20	0.01	0.33
7	0.59	0.71	0.42	0.72	0.78	0.48	0.64	0.86	0.11
8	0.57	0.65	0.42	0.63	0.78	0.27	0.65	0.75	0.42
9	0.58	0.72	0.51	0.72	0.82	0.48	0.67	0.82	0.49
10	0.65	0.78	0.65	0.72	0.80	0.17	0.57	0.68	0.43
11	0.60	0.76	0.60	0.71	0.79	0.71	0.58	0.73	0.22
12	0.63	0.79	0.63	0.74	0.82	0.74	0.61	0.74	0.39
Mean	0.61	0.74	0.49	0.70	0.79	0.45	0.58	0.68	0.33

*DSC* Dice similarity coefficient, *PV* portal vein, *HV* hepatic vein, *BD* bile duct.

## Discussion

This study highlights the potential of 3D residual U-Net model for automatic segmentation of the liver and its intrahepatic structures from single-phase MRI images, even when facing a challenging task of segmenting five distinct structures (liver parenchyma, tumor mass, hepatic vein, portal vein, and bile duct) in the medical domain. Importantly, MRI data in this study were acquired from patients with intrahepatic masses, which deviated from normal anatomy. Nevertheless, the model showed high accuracy for predicting the liver parenchyma (DSC: 0.92 ± 0.03) and tumors (DSC: 0.77 ± 0.21).

The primary aim of this study was to enhance our understanding of the spatial relationship between tumors and major hepatic vasculatures. For surgeons performing liver resection, knowledge for the anatomical relationship is crucial, as it influences the plane of resection, resultant tumor margin, remnant liver volume, and the decision to perform anatomic or non-anatomic resection^7^. However, most deep learning studies on liver imaging have focused on segmenting gross anatomical structures such as the liver parenchyma and tumors^8,9^. While some studies have aimed at predicting liver fibrosis or evaluating fatty change, they do not offer comprehensive information for surgeons in their preoperative planning for liver resection procedures.

Hepatic vasculatures exhibit linear and tubular morphology, and these characteristics can significantly reduce segmentation accuracy with even minor prediction errors when evaluated using DSC as a metric^10^. Nonetheless, our study achieved a DSC of 0.70 ± 0.05 for HV and 0.61 ± 0.03 for PV, indicating a satisfactory performance despite challenges posed by these structures. A previous study has segmented three classes and reported a DSC of 0.93 for liver parenchyma, 0.53 for HV, and 0.64 for PV from non-contrast T1 vibe Dixon sequence MRI^11^. Our study achieved a higher DSC for HV with similar results for parenchyma and PV. Moreover, our study included five classes and involved patients with abnormal anatomical structures due to intrahepatic tumors. Another study has segmented hepatic vasculatures with a high accuracy using gadoxetic acid-enhanced multi-phase MRI images and evaluated performance using F1, precision, and sensitivity metrics^12^. In contrast, our study used only single-phase MRI and involved segmenting five classes, including tumors, a distinguishing feature from others.

We opted to use the 20-min delayed hepatobiliary phase gadoxetic acid-enhanced liver MRI because it effectively displayed all structures crucial for surgery within a single phase, providing numerous benefits for multi-structure segmentation. The hepatobiliary phase is the optimal sequence for clearly illustrating the distinction between liver parenchyma and tumors. At 20 min after contrast infusion, the liver parenchyma exhibits relatively high enhancement, while the tumor mass displays lower enhancement, enabling differentiation from liver parenchyma^13,14^. Concurrently, vascular structures such as portal and hepatic veins present low intensity, with biliary structures being the highest intensity due to contrast excretion through the bile duct. Among all MRI sequences, the 20-min delayed hepatobiliary phase is the only phase capable of accurately delineating these five structures. Utilizing a single-phase multi-structure segmentation approach can streamline clinical application by circumventing registration errors commonly associated with merging data from multiple sequences.

From a surgeon’s perspective, planning hepatic resections using the Glissonean pedicle approach necessitates a thorough understanding of the main branch of the portal vein’s anatomical structure around the hilar plate^15^. Moreover, accurate comprehension of the main branch anatomy for both portal and hepatic veins is essential for preventing post-hepatectomy liver failure and preserving an adequate future remnant liver volume^16^. To further assess our model’s performance, we separately calculated DSCs for main and peripheral branches of the hepatic vein, portal vein, and bile duct as illustrated in Table 3. Findings revealed that focusing solely on the main branch of each structure yielded higher DSC values. This implies that the accuracy of peripheral branches is relatively lower compared to that of main branches. However, the significance of these peripheral branches is less critical than that of major branches, rendering our deep learning model suitable for surgeons preparing surgical plans.

Limitations of this study include its single-center design and the relatively small size of dataset, which might restrict the generalizability of our findings. While this approach was chosen to maximize efficacy with limited resources, future research should aim to collect diverse dataset from multiple institutions to develop a more robust and widely applicable model. Furthermore, our study’s patient data were primarily focused on those with hepatic masses scheduled for liver resection, potentially limiting the model’s validation for cases with disseminated, inoperable masses. Additionally, our study utilized 20-min delayed gadoxetic acid-enhanced MRI, which might yield lower image quality in patients with severe liver cirrhosis, making it challenging to distinguish nodular hepatic parenchyma from vascular structures. Consequently, accuracy might be relatively lower for these patients. Lastly, this study solely employed the Dice similarity coefficient as a performance evaluation metric without considering other metrics such as sensitivity or specificity.

Liver surgeons can benefit from three-dimensional reconstruction of preoperative liver images. Research in this area continues to advance alongside technological developments^3,17,18^. While products are available on the market^19^, the unmet need has not been fulfilled by their high costs, with cost-effectiveness being a concern. The cost and effort for utilizing the technology only permit high volume centers to use it, especially in regions like Republic of Korea, where 3D reconstruction technology is not covered by the national insurance system. Additionally, as 3D reconstruction is not fully automated, there is a disadvantage in terms of human time cost. However, our study introduces an automatic segmentation model that utilizes deep learning. It offers the advantage of using only a single-phase MRI, reducing human time required for the task. While our deep learning model needs further improvement for practical use, we consider that the goal is near with an intuitively acceptable outcome of our 3D reconstructed models.

## Methods

### Study population

In this single-institution retrospective study, adult patients (age > 18 years) who underwent liver resection due to a hepatic mass between January 2020 and December 2022 were included. This study included patients who had preoperative gadoxetic acid enhanced MRI, the raw image data for deep learning model training. Demographic and clinical characteristics such as age, sex, body mass index, diagnosis, tumor size, and number were collected. Data of included patients were extracted from the Clinical Data Warehouse DARWIN-C of Samsung Medical Center. This study was approved by the institutional review board of Samsung Medical Center (SMC 2023-02-049) and the need for informed consent was waived by the IRB due to the retrospective nature of the study. It was carried out in accordance with the principles of the Declaration of Helsinki.

### Dataset

This study used hepatobiliary phase images from gadoxetic acid enhanced MRI from each patient as this phase showed the best liver contour, hepatic mass, hepatic vein, portal vein, and bile duct. To obtain the dataset, we used 3.0-T MRI machines (Archieva or Ingenia, Philips Healthcare, Best, the Netherland) and performed liver MRI examination including the hepatobiliary phase using protocols shown in Supplementary Material 1.

**Figure 2:**
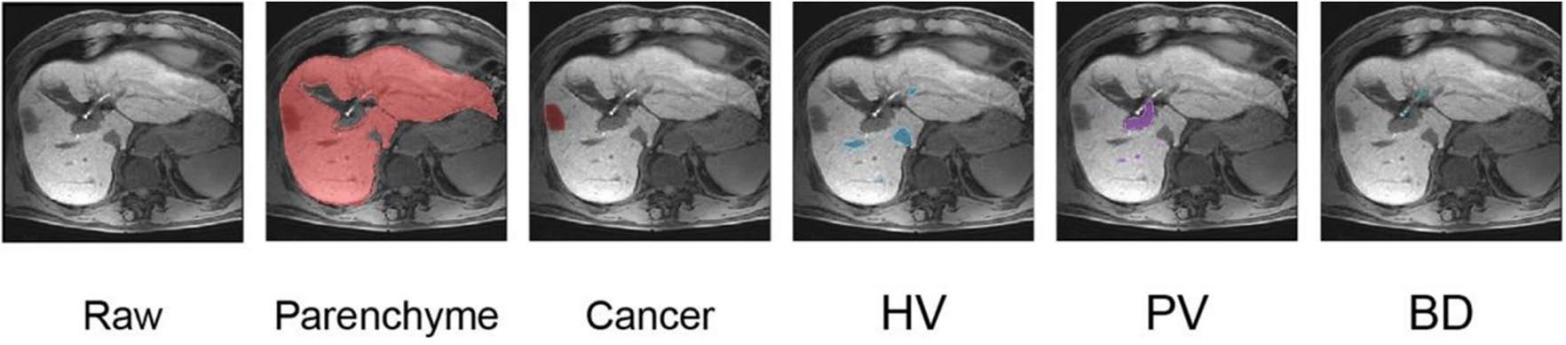
Manual segmentation of liver MRI for this study. Examples of slice-by-slice manual delineation of liver parenchyma, cancer, hepatic vein (HV), portal vein (PV), and bile duct (BD) by trained biomedical visualization artists are shown. They are confirmed by an abdominal radiologist and a hepatic surgeon.

Liver MRI datasets were manually labelled slice-by-slice for the outer liver border, margin of tumor, portal vein, hepatic vein, and bile duct by two trained biomedical visualization artists. Results were confirmed by a board-certified abdominal radiologist and hepatic surgeon (Fig. 2). In case of disagreement, a consensus was reached through discussion. A total 120 patients were included, and the training and validation sets were chosen using random sampling. Liver MRIs from 108 patients were used for training (training set) and those from 12 patients were used for validation (validation set).

### 3D residual U-Net model

**Figure 3:**
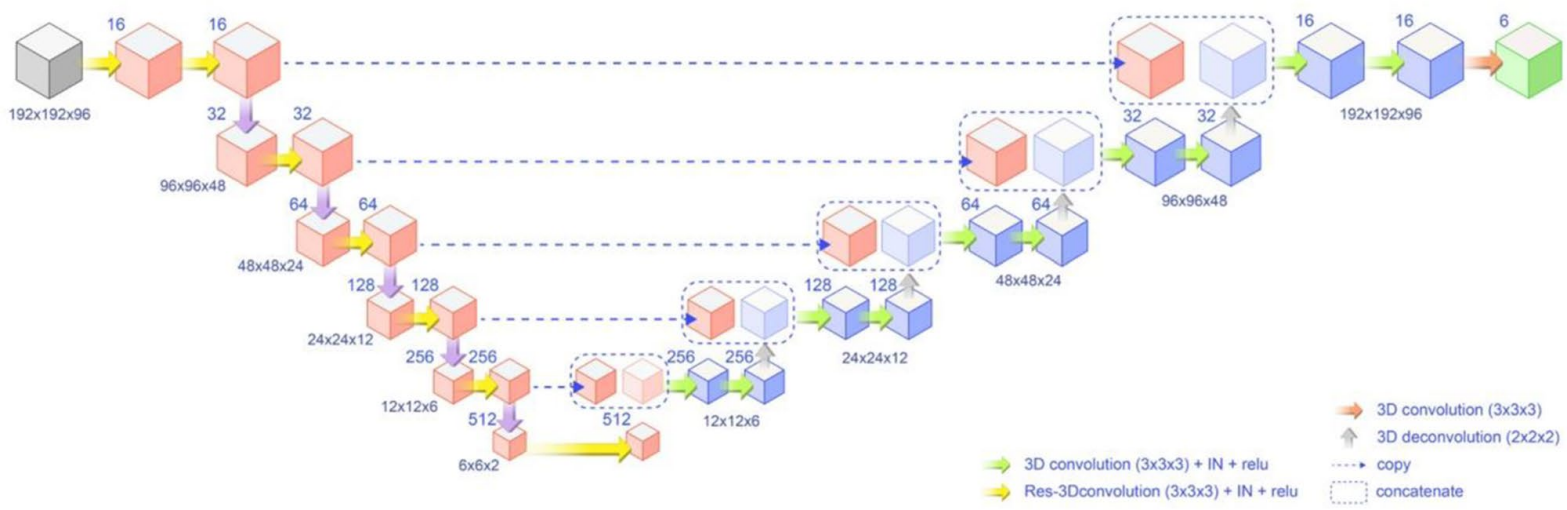
Schematic representation of the 3D residual U-Net model. The model demonstrates encoding and decoding processes, with convolutional residual operations during encoding and multi-target segmented image reconstruction using deconvolutional feature maps during decoding. Skip connections are shown at each resolution level.

We utilized a 3D residual U-Net model for this study (Fig. 3). The model could extract context features through an encoding process and use these features to reconstruct segmented images during decoding. The model was trained to minimize loss function, optimizing network parameters for hierarchical feature extraction. During encoding, convolutional residual operation was employed. During decoding, multi-target segmented images were reconstructed with deconvolutional feature maps, including skip connections at each resolution level.

### Implementation

The 3D residual U-Net model was implemented in TensorFlow 1.14 and trained on a workstation with four GPUs (NVIDIA TITAN XP 16 GB). During preprocessing, images were cropped, resized, and normalized. Training data were augmented through various techniques, such as 3D rotation, scaling, random flipping, and cropping. Dice similarity coefficient loss (L) was used as the loss function. The network was trained using the Adam optimizer at a learning rate of 0.0001, with 1000 epochs and a batch size of 4. During the testing phase, preprocessed images of the entire hepatobiliary phase MRI scan were fed into the proposed network. For more detailed information on the 3D residual U-Net model and implementation, please refer to Supplementary Material 2.

### Evaluation

The performance of the deep learning model for segmenting liver parenchyma, tumor, portal vein, hepatic vein, and bile duct was compared with manually delineated liver parenchyma, tumor, portal vein, hepatic vein, and bile duct, respectively. Dice similarity coefficient (DSC) was used to quantify segmentation performance of the deep learning model. For visualization, all structures including liver parenchyma, tumor mass, hepatic vein, portal vein, and bile duct were 3-dimensionally reconstructed with Mimics Medical (Materialise, Leuven, Belgium).

### Statistical analysis

Continuous variables were presented as mean ± standard deviation and analyzed using the independent t-test or Mann–Whitney test, as appropriate. Categorical data were presented as numbers and percentages and analyzed using the Chi-squared or Fisher’s exact test. All statistical analyses were performed using R Statistical Software (version 3.6.3; Foundation for Statistical Computing, Vienna, Austria).

### Supplementary Information

Supplementary Information.

## Data Availability

Data generated or analyzed during the study are available from the corresponding author by request.

## Abbreviations

AI: Artificial intelligence
BD: Bile duct
CT: Computed tomography
DSC: Dice similarity coefficient
FDA: Food and drug administration
GPU: Graphics processing unit
HCC: Hepatocellular carcinoma
HV: Hepatic vein
MRI: Magnetic resonance imaging
PV: Portal vein
