# Supplementary material for: Automated 3D liver segmentation from hepatobiliary phase MRI for enhanced preoperative planning
Source: Sci Rep. 2023 Oct 17;13:17605. doi: 10.1038/s41598-023-44736-w (PMC10582008; doi:10.1038/s41598-023-44736-w)
Supplement: Supplementary file 1 — Supplementary Information. [file 41598_2023_44736_MOESM1_ESM.docx]

**Supplementary 1. Detailed protocol of liver MRI examination**

sequence = 3D T1-weighted imaging in 20 minutes after gadoxetic acid injection; repetition time (TR)/echo time (TE) = shortest (around 4 msec)/shortest (0 msec); Flip angle = 10 degree; fat suppression method = Spectral Adiabatic Inversion Recovery (SPAIR) or modified DIXON; inversion time = shortest; matrix size = 320×256; in-plane resolution = 1.17 mm; number of signal acquisition = 1; echo train length = 2; slice thickness = 4 mm; slice spacing = 2 mm; and number of slices = 100 images.

**Supplementary 2. 3D Residual U-Net model and implementation**

The 3D residual U-Net model was depicted in Figure 3. The 3D residual U-Net extracts context features through an encoding process and uses these features for reconstructing the segmented image through the decoding process.

Given a training set ($S$) of images and labels $S=\left\{ \left( I_{n},G_{n} \right), n=1,\ldots,N \right\}$, where $I_{n}$ denotes a MRI image and $G_{n}$ indicates the ground truth label of the image, the model can be trained to minimize a loss function $\mathcal{L}$ in order to optimize the model $f(I,\Theta)$, where $\Theta$ denotes the network parameters that include the convolutional kernel weights for hierarchical feature extraction.

During encoding, the convolutional residual operation is employed. In this paper, the convolutional residual operation is used as follows:

$x_{l+1}=relu\left( bn\left( conv\left( x_{l} \right) \right) \right)+x_{l}$, (1)

where $x_{l}$ is the output of the $l^{th}$ layer and $conv(\cdot)$ is a convolution function followed by a batch normalization $bn(\cdot)$ + rectified linear unit (ReLU) $relu\left( \cdot\right)$. In residual network, as we redefine the mapping function containing residual operation to $h_{l}$, the output of the previous layer $x_{l-1}$, $x_{l}$ denote as

$x_{l+1}=h_{l}(x_{l})$. (2)

During decoding, multi-target segmented images were reconstructed with deconvolutional feature maps by concatenating the encoder’s feature maps as shown in Figure 3. At each resolution level, a skip connection is included to concatenate the up-sampled feature maps with the same level feature maps obtained from both encoders.

Let $d^{2}(\cdot)$ denote the deconvolution function with stride 2. The up-sampled feature maps are expressed as:

$x_{l+1}=relu(bn(conv\left( deconv\left( x_{l} \right)\circ x_{t} \right))),$ (7)

where $\circ$ indicates the concatenation and $t$ shows the same resolution level in encoders. The final output of the decoder performs 1×1×1 convolution and reduces the number of output channels to the number of class labels (six outputs; liver parenchyma, liver tumor, portal vein, hepatic vein, bile duct and background).

The 3D residual U-Net model was implemented in TensorFlow 1.14. Training and testing of the proposed network were performed on a workstation with four GPUs (NVIDIA TITAN XP 16GB). During preprocessing, a voxel size of 1.0 x1.0 x 2.0 mm^3^ is considered. The MR intensity value was normalized using mean and standard deviation in 3D MRI. The normalized images, which were used as inputs of the model, were cropped with padding, resulting in a dimension of 256×256×128.

For better generalization of trained networks, the training data was augmented through 3D rotation of each axis with -15 to 15 degrees, 3D scaling from 0.85 to 1.15, random flipping of each axis during training, and randomly cropping the preprocessed image as 192×192×96 patches for the input of network during training.

The dice similarity coefficient loss ($\mathcal{L}$) was used as the loss function followed by

$\mathcal{L}\left( X,\Theta,G \right)=-\frac{1}{K}\sum_{k=1}^{K} \left( \frac{2\sum_{i}^{N} p_{i,k}g_{i,k}}{\sum_{i}^{N} p_{i,k}+\sum_{i}^{N} g_{i,k}} \right)$ (6)

where $p_{i,k}\in[0,\ldots,1]$ represents the continuous values of the $softmax$ 3D prediction maps for each class label $k\in[0,1,2, 3, 4, 5]$ and $g_{i,k}\in[0,1]$ the corresponding ground truth value at each voxel $i$. Here, the number of classes is set to 6: liver parenchyma, liver tumor, portal vein, hepatic vein, bile duct and background. The network was trained using the Adam optimizer with a learning rate of 0.0001 by end-to-end learning. The number of epochs was set to 1000 and the size of the batch was set to 4. In the testing phase, the preprocessed images of the entire MRI scan were fed into the proposed network.
